# Impact of the Synthesis Method on the Conventional and Persistent Luminescence in Gd_3–*x*_Ce_*x*_Ga_3_Al_2_O_12_

**DOI:** 10.1021/acs.inorgchem.1c02239

**Published:** 2021-12-01

**Authors:** Paweł Głuchowski, Kamila Rajfur

**Affiliations:** † Institute of Low Temperature and Structural Research PAS, PL-50422 Wroclaw, Poland; ‡ Wroclaw University of Science and Technology, PL-50370 Wroclaw, Poland

## Abstract

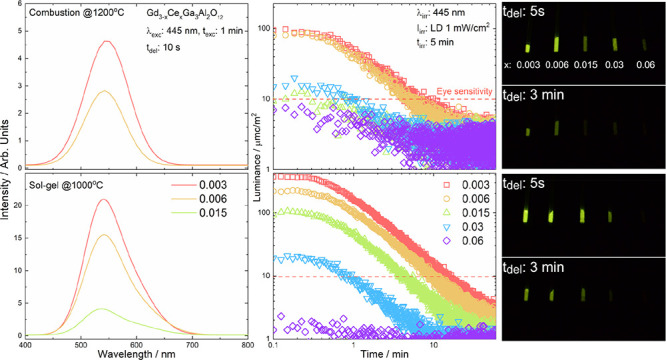

The series of Gd_3–*x*_Ce_*x*_Ga_3_Al_2_O_12_ nanopowders doped with different concentrations of Ce^3+^ ions were prepared by Pechini (sol–gel) and combustion methods. The structure and morphology of the powders were characterized by X-ray diffraction (XRD) and scanning electron microscopy (SEM) techniques. It was found that the synthesis method has a great impact on the morphology and, consequently, spectroscopic properties of the powders. Optical properties of the powders were examined using excitation, emission, and luminescence kinetic measurements. For all powders, persistent luminescence and emission decay processes were studied. The most intense luminescence was observed for the powder with 0.5 mol % of Ce^3+^ synthesized using the combustion method and 1 mol % in the case of the sol–gel sample. The longest and brightest persistent luminescence was observed for the powders doped with 0.1 mol % (combustion) and 0.2 mol % of Ce^3+^ ions (sol–gel). The thermoluminescence measurements were done for the powders prepared using different methods to understand the impact of the synthesis conditions on the number and depths of the traps involved in persistent luminescence. On the basis of spectroscopic measurements, the mechanism of persistent luminescence was constructed and discussed.

## Abstract 2

The impact of different synthesis methods and Ce^3+^ concentration on the structure and spectroscopic properties of Gd_3−*x*_Ce_*x*_Ga_3_Al_2_O_12_ powders was studied. The longest and most intense emission was observed for the lowest Ce^3+^ concentration. For high dopant concentration, persistent luminescence was not observed due to the increase of the number of relaxation centers near electron traps. It was also shown that the number and location of the traps are strongly affected by the synthesis method.

## Introduction

The phenomenon of persistent luminescence describes the release of energy stored by lattice defects located near the conduction band (CB). Furthermore, it is also interesting from a practical point of view and is commonly used in many different areas of applications such as emergency signaling,^1,2^ biolabeling^3−5^ or the creation of luminophores for white LEDs,^6,7^ just to name a few. Persistent luminescence is usually observed only at room temperature^8^ since, at higher temperatures, the stored energy is rapidly released, thus reducing the quality and efficiency of the phosphor. Depending on the phosphor composition, the effect can last from just a few seconds up to several hours.^9^ The most common color of emitted light is green,^10^ but blue,^11^ yellow,^12^ orange,^13^ or red/NIR^14−16^ persistent luminophores have also been obtained by researchers. The development of phosphors with emission in the red/NIR region has attracted even more attention since it is based mainly on the use of these materials in various fields of biology and medicine. This is due to the fact that the absorption of biological tissues in this range is significantly lower than in the remaining part of the visible spectrum.^17^

Persistent luminescence has been mainly observed in oxides,^9,18^ sulfides,^19,20^ and nitrides.^21−23^ However, due to the wide possible choice of crystal structure, elemental composition, chemical stability, and the possibility of tuning the electronic structure, most research today is focused on the oxide materials. Especially extensive research is being carried out on the group of gallates^24,25^ and gallogermanates^26,9^ doped or co-doped with Cr^3+^ ions due to the fact that they exhibit long and bright emission in the red/NIR range. Additionally, due to the easily modifiable electronic structure (band gap width and crystal field strength), the matrices from the group of garnets are also very popular for persistent luminescence applications and research, mainly YAGG^27,28^ and GGAG^29^ compounds since they have great potential in band gap engineering and allow for the creation of structures with desired electronic properties, for which positions of the energy traps or excited states of dopants in the band gap can be easily controlled (e.g., in the conduction band or below it).^30,31^

Garnets doped with cerium are most often studied for use as phosphors for white LEDs^32,33^ and, thanks to the short luminescence decay times, also as scintillators.^34,35^ These studies are focused on obtaining structures with high quantum efficiency,^36^ appropriate emission color (high color-rendering index, CRI), and high temperature stability.^37^ Various external factors can affect the splitting of the excited 5d levels of Ce^3+^ ions and, in turn, have a great impact on their spectroscopic properties. Multiple different studies have shown that, depending on the type of garnet composition, the optimal concentration of Ce^3+^ ions for efficient luminescence ranges from 0.3 to 6 atom %^37−39^ with concentration luminescence quenching for a higher dopant level. At the same time, the concentration quenching effect for persistent luminescence was studied only in a relatively small number of papers.^40^

The aim of the present work is to investigate and understand the persistent luminescence quenching effect in Gd_3–*x*_Ce_*x*_Ga_3_Al_2_O_12_ prepared by two different synthesis procedures. Powders with various doping levels were prepared to determine the Ce^3+^concentration high enough for emission quenching to occur. Overall, the temperature quenching of Ce^3+^ luminescence in Gd_3_Ga_3_Al_2_O_12_ is low due to the high ionization energy of Ce^3+^ ions in the matrix. The energies of the host CB and Ce^3+^ 5d levels play a critical role in determining the optimal doping level to obtain efficient and long persistent luminescence. Lastly, it was observed that the optimal concentration of Ce^3+^ is much lower for persistent emission than that for conventional luminescence.

## Experimental Section

The powders were synthesized using sol–gel (Pechini)^41^ and combustion methods.^42^ To obtain materials with different concentrations of cerium, the stoichiometric amount of gadolinium oxide (Gd_2_O_3_, 99.9%, Onyxmet, Poland) was dissolved in diluted nitric acid and deionized water. Solutions were evaporated and dissolved in deionized water again three times to obtain pure nitrate. Gallium, aluminum, and cerium ions were added in the form of hydrated nitrates Ga(NO_3_)_3_·H_2_O (99.9%, Onyxmet, Poland), Al(NO_3_)_3_·9H_2_O (98.0–102.0%, ACS, USA), and Ce(NO_3_)_3_·6H_2_O (99.99%, Sigma Aldrich, USA). The citric acid (99.5%, anhydrous, ACS, USA) was added as a chelating agent to form polybasic acid chelates with cations, and then ethylene glycol (CZDA, POCH, Poland) was added to start the polyesterification of the solution. After 3 h of stirring, the solution was dried in 90 °C for a few days until a brown resin was formed. The resin was taken to the crucibles, calcined in air at 1000 °C for 8 h, and grinded in an agate mortar into powders. Another approach to obtain GGAG powders was based on the combustion method. The first stage of the synthesis was the same as for the sol–gel one. Gadolinium oxide was dissolved in nitric acid, and by recrystallization three times, pure nitrate was obtained. Then the nitrates of gallium, aluminum, and cerium were added. Urea was added to the solution as a fuel in the molar ratio of 15 mol of fuel/reducer for 6 mol of each nitrate/oxidizer. Solution was evaporated and then placed in the furnace preheated to 650 °C. Self-propagated combustion took place in air atmosphere during several seconds. The samples stayed for 5 min in the furnace and then were taken for grinding. Samples prepared using the combustion method were divided into two parts, and one of them was calcined again in the air at 1200 °C for 6 h.

## Equipment

The structure of the samples was studied by an X’PERT PRO PANalytical diffractometer (Malvern Panalytical, Almelo, The Netherlands) using copper Kα_1,2_ radiation (λ = 0.15418 nm) in the 2Θ range from 10 to 80°. A scanning electron microscope (SEM; FEI Nova NanoSEM 230 (USA)) was used to reveal the crystallite size and the morphology of powders prepared by different methods. The homogeneity of the powders was performed using the scanning electron microscope (FESEM FEI Nova NanoSEM 230) equipped with an EDS spectrometer (EDAX Genesis). The excitation and emission spectra were recorded using an FLS980 Fluorescence Spectrometer (Edinburgh Instruments) equipped with holographic grating (1800 lines/mm), blazed at 300 mm focal length monochromators in Czerny Turner configuration. The excitation and emission spectra were obtained using a 450 W Xenon lamp. The persistent luminescence was measured using a SILVER-Nova Super Range TE Cooled Spectrometer (StallarNet Inc.) with 200 μm slit and 445 nm CNI laser diode (2500 mW) as an excitation source. To prevent heating of the samples, the power of the excitation source was limited to 750 mW. The samples were irradiated for 5 min, and the persistent luminescence spectra were recorded 5 s after switching off the excitation. The persistent luminescence fading curves were monitored using a Jobin Yvon Spectrometer equipped with a Hamamatsu R928 photomultiplier. The thermoluminescence was detected by a Lexsyg Research Fully Automated TL/OSL Reader (Freiberg Instruments GmbH) for each sample previously irradiated by the 445 nm CNI laser diode (2500 mW) at the same conditions. The TL glow curves were collected with an R13456 photomultiplier tube (Hamamatsu Measurements) for the powders sprayed on the sample holder. The TL curves were recorded from 300 to 600 K at the heating rate of 5 K/s. The XPS analyses were carried out with a Kratos Axis Supra spectrometer using a monochromatic Al Kα source (10 mA, 15 kV). The instrument work function was calibrated to give a binding energy (BE) of 83.96 eV for the Au 4f7/2 line for metallic gold, and the spectrometer dispersion was adjusted to give a BE of 932.62 eV for the Cu 2p_3/2_ line of metallic copper. High-resolution analyses were carried out with an analysis area of 300 × 700 μm and a pass energy of 20 eV. Spectra have been charge corrected to the main line of the carbon 1s spectrum (adventitious carbon) set to 284.8 eV. Spectra were analyzed using the CasaXPS software (version 2.3.23rev1.1R).

## Results and Discussion

### Structure and Morphology of the Samples

**Figure 1:**
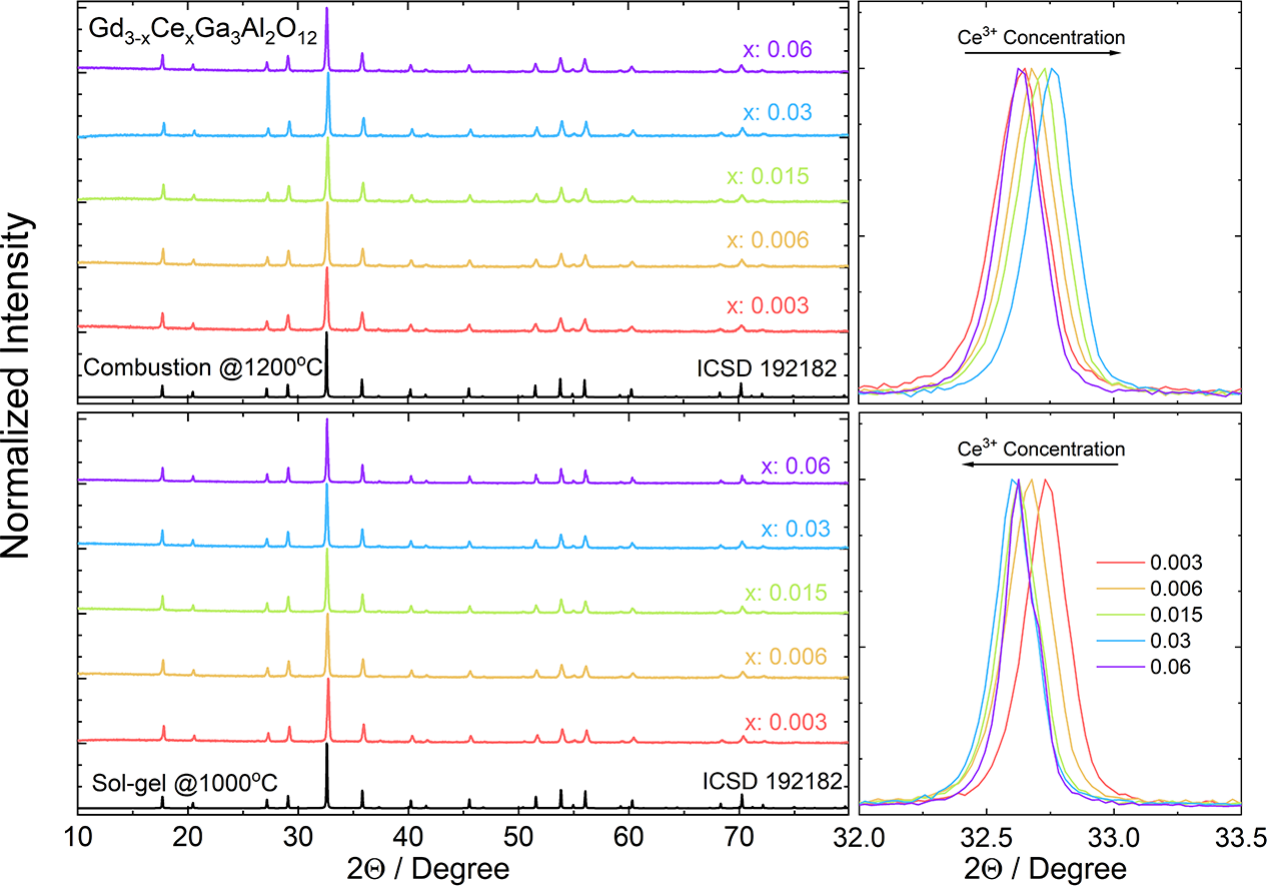
X-ray diffraction patterns of Gd_3–*x*_Ce_*x*_Ga_3_Al_2_O_12_ prepared using combustion with additional calcination (top) and sol–gel (modified Pechini) methods (bottom).

X-ray powder diffraction results for the powders obtained by the combustion and the sol–gel method are shown in Figure 1. It can be seen that all reflections for powders annealed at high temperatures (sol–gel and combustion methods with additional calcination) correspond to the garnet structure of the Gd_3_Ga_3_Al_2_O_12_ (ICSD 192182). X-ray diffraction patterns show that obtained materials crystallize in the cubic crystal structure with the *Ia*3̅*d* space group (*Z* = 8). For the powders obtained by the combustion method without additional calcination, the pronounced peak at ≈32.5° is split, and also, the baseline for all diffraction patterns is raised, suggesting that part of the material was not fully crystallized. The XRD data correspond well to the garnet structure even at the highest doping level due to similar ionic radii of Gd^3+^ (0.938 Å) and Ce^3+^ (1.01 Å) occupying its position.^43^ Although the structure agrees well with the reference pattern, one can observe that with the change in Ce^3+^ concentration, the peaks are shifted toward lower (sol–gel) or higher (combustion) angles. The change in the position of diffraction peak indicates an enlargement or reduction of a unit cell volume. So, the unit cell increases with increasing Ce^3+^ concentration for the combustion method and decreases with increasing Ce^3+^ concentration the for sol–gel method. For the samples obtained by the combustion method with a much wider crystallite size distribution (e.g., crystallites larger than a few micrometers are observed), the impact of Ce^3+^ concentration on the unit cell size is different, so for the highest concentration, the change of unit cell size does not follow the trend observed for the rest of the samples. Probably, this difference is due to the diffusion process and a possible segregation of the dopant not detected by X-ray diffraction. So, for both methods, the dopant concentration is of great importance for the course of the reaction. During the combustion process, nitrates act as an oxidizing agent promoting a rapid increase of the temperature and taking an active role in the initial phase of the synthesis involving a violent reaction and fast crystal growth. This can result in the simultaneous formation of large micron and small nano-sized crystals with a wide size distribution. In case of the sol–gel method, nitrates do not participate directly in the reaction because they are cross-linked in polymer chains and the annealing temperature changes slowly. This feature has a great impact on the processes of nanocrystal growth, dopant segregation in grain boundaries, and formation of the oxygen vacancies taking part in the creation of the energy traps.

The results of Rietveld analysis for XRD patterns of the powders (X’PERT PRO analysis software^44^) are shown in Table 1. One can observe that, with an increase of Ce^3+^ concentration, the crystallite size increases slightly. Also, in the case of the combustion method, the volume of the unit cell increases with an increase of Ce^3+^ concentration, and for the sol–gel method, the tendency is opposite. The lattice strains change irregularly and cannot be directly related to the change in the dopant concentration.

**Table 1:** Crystallographic Parameters of Gd_3–*x*_Ce_*x*_Ga_3_Al_2_O_12_ Refined Using Rietveld Analysis for Powders Prepared by the Combustion with Additional Calcination and Sol–Gel (Modified Pechini) Methods

*x*	*R*_exp_, GOF^a^	crystallite size (nm)	unit cell size (Å)	strain (%)	volume (Å^3^)
Combustion @ 1200 °C					
0.003	1.658, 1.843	49	12.274(7)	0.022	1849.4
0.006	1.925, 2.221	53	12.274(9)	0.021	1849.5
0.015	2.032, 2.372	52	12.279(9)	0.001	1851.8
0.03	1.889, 1.926	59	12.278(6)	0.023	1851.2
0.06	1.984, 2.104	66	12.274(3)	0.002	1849.2
Sol–Gel @ 1000 °C					
0.003	1.449, 1.732	64	12.273(2)	0.019	1848.8
0.006	1.643, 1.933	58	12.272(5)	0.001	1848.4
0.015	1.561, 1.796	74	12.268(6)	0.000	1846.7
0.03	1.599, 1.889	74	12.270(6)	0.016	1847.6
0.06	1.475, 1.745	129	12.269(1)	0.000	1846.9

a *R*_exp_, expected Rietveld *R* factor; GOF, goodness of fit.

Ce^3+^ ions substituting Gd^3+^ cations occupy the dodecahedral [A] sites of the [A]_3_[B]_2_[C]_3_O_12_ cubic garnet structure.^45^ The bond lengths between Gd^3+^/Ce^3+^ and oxygen ions and between oxygen ions forming an edge of the dodecahedral site (with four octahedral and six tetrahedral sites) have a great impact on the spectroscopic properties of Ce^3+^ ion. For this reason, the bond length between Gd^3+^/Ce^3+^ and oxygen ions was calculated using Rietveld analysis (Table 2). It can be seen that for powders obtained by the combustion method, the changes are very small and irregular. In the case of the sol–gel method, the bond length shortens with increasing Ce^3+^ ion concentration.

**Table 2:** Gd^3+^/Ce^3+^–O^2–^ Bond Lengths Calculated for Powders Obtained by Two Different Methods

	combustion @ 1200 °C		sol–gel @ 1000 °C	
*x*	Gd/Ce–O_OS_ (Å)	Gd/Ce–O_TS_ (Å)	Gd/Ce–O_OS_ (Å)	Gd/Ce–O_TS_ (Å)
0.003	2.5202	2.4184	2.5198	2.4180
0.006	2.5201	2.4183	2.5195	2.4178
0.015	2.5208	2.4189	2.5187	2.4169
0.03	2.5203	2.4186	2.5191	2.4173
0.06	2.5198	2.4180	2.5185	2.4168

**Figure 2:**
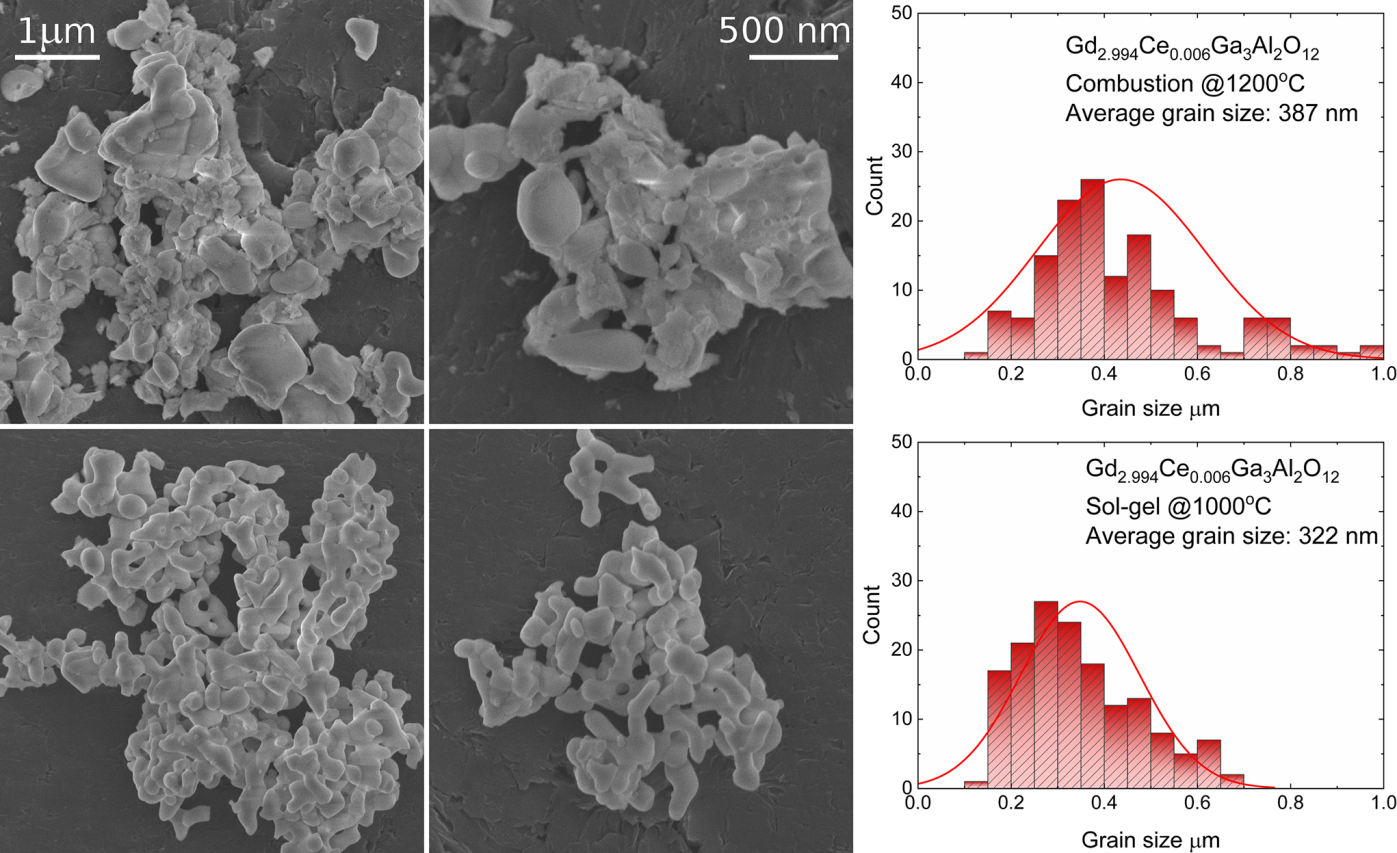
SEM images and grain size distribution of Gd_2.994_Ce_0.006_Ga_3_Al_2_O_12_ obtained using combustion (top) and sol–gel (bottom) methods.

For two representative powders obtained by combustion with additional calcination and sol–gel methods, SEM images were taken to reveal the impact of the synthesis conditions on the morphology of the grains (Figure 2). It can be observed that, for the combustion method, grains are more irregular and have a broader crystallite size distribution, with a higher average grain size. The powders are composed of small crystallites with the sizes of tens of nanometers, but microsized crystals are also clearly observed. For the powders obtained by the sol–gel method, the grains are smoother and exhibit a narrower size distribution. Most of the crystallites have an oblong, oval shape. It can be seen that some of the bars stuck together under the influence of high temperature, creating more complex spatial structures, but their size is still under a micrometer. As the powders should undergo ceramic sintering, a regular shape is highly desirable for easier organization and arrangement into regular structures under the influence of high pressure.^46^

**Figure 3:**
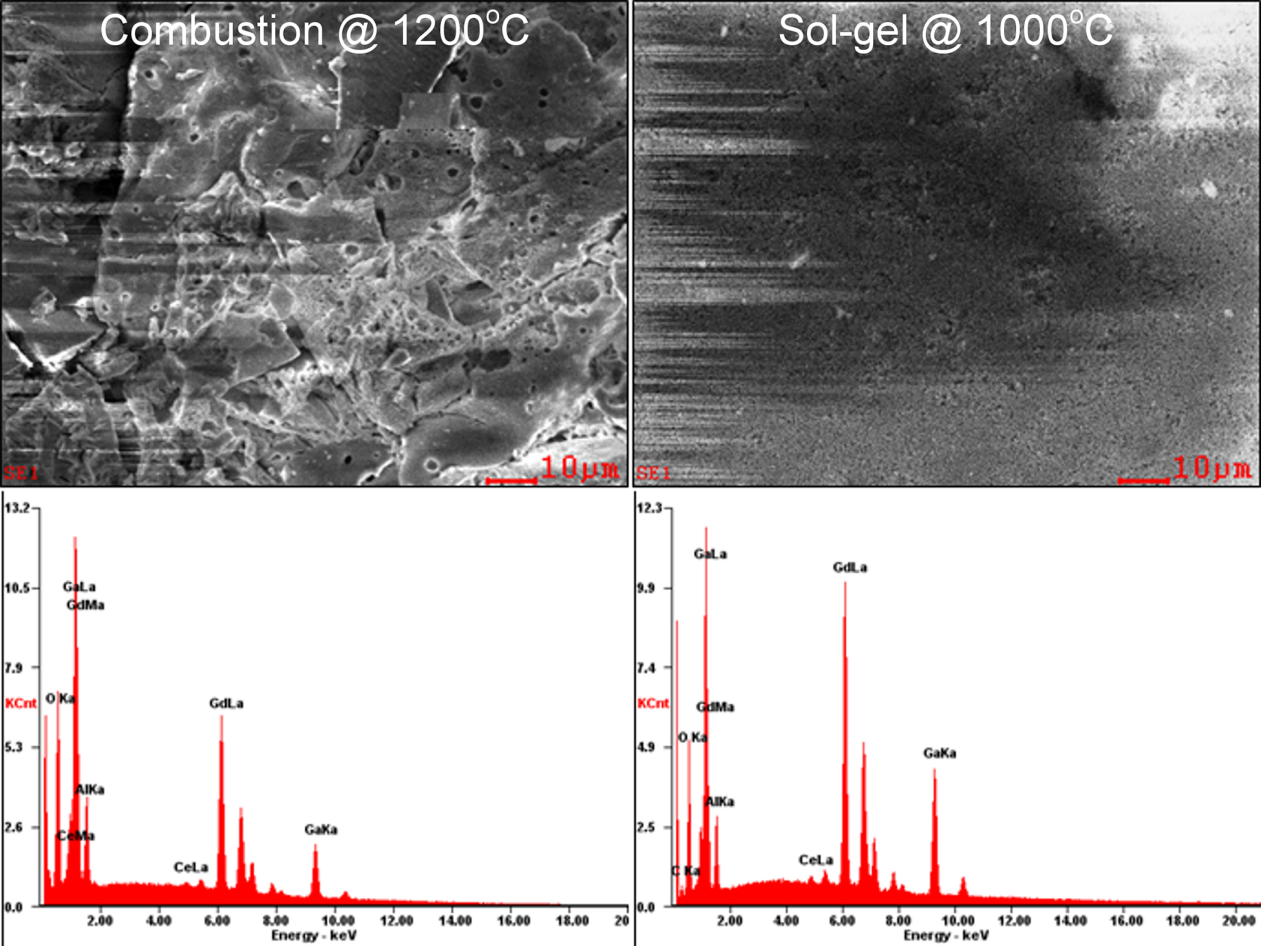
EDS spectra of Gd_2.94_Ce_0.06_Ga_3_Al_2_O_12_ obtained using combustion (left) and sol–gel (right) methods.

For Gd_2.994_Ce_0.006_Ga_3_Al_2_O_12_ powders obtained using combustion and sol–gel methods, the energy dispersive spectroscopy (EDS) maps were prepared to check the elements’ distribution (Figure 3). The EDS analyses were performed at 20.0 kV from the large area (250 μm × 200 μm) of the samples. The powder samples were included in the carbon resin and then pressed to obtain a large and flat area. Signals from three randomly selected areas were collected to ensure satisfactory statistical averaging. It was not possible to perform the measurement for the sample containing the smallest amount of Ce^3+^ with the appropriate accuracy; therefore, this result was omitted in Table 3. The quantitative analysis accuracy for standardless analysis where results are below 1 wt % is burdened with a high error (even up to 50%), but despite the high error, the results show a good agreement of the obtained results with the assumed values of the concentration of ions used in the synthesis (Table 3).

**Table 3:** EDS Analysis of Ce^3+^ Concentration in the Gd_3–*x*_Ce_*x*_Ga_3_Al_2_O_12_ Powders Obtained by Two Different Methods

	Ce^3+^ concentration (atom %)							
	combustion @ 1200 °C				sol–gel @ 1000 °C			
*x*	1	2	3	avg	1	2	3	avg
0.006	0.47	0.57	0.57	0.54	0.44	0.25	0.4	0.36
0.015	0.7	0.45	0.49	0.55	0.53	0.58	0.57	0.56
0.03	1.29	0.94	1.01	1.08	0.85	0.88	1.02	0.92
0.06	2.22	2.02	1.93	2.06	2.04	2	2.05	2.03

### Excitation and Emission Spectra of Gd_3–*x*_Ce_*x*_Ga_3_Al_2_O_12_

**Figure 4:**
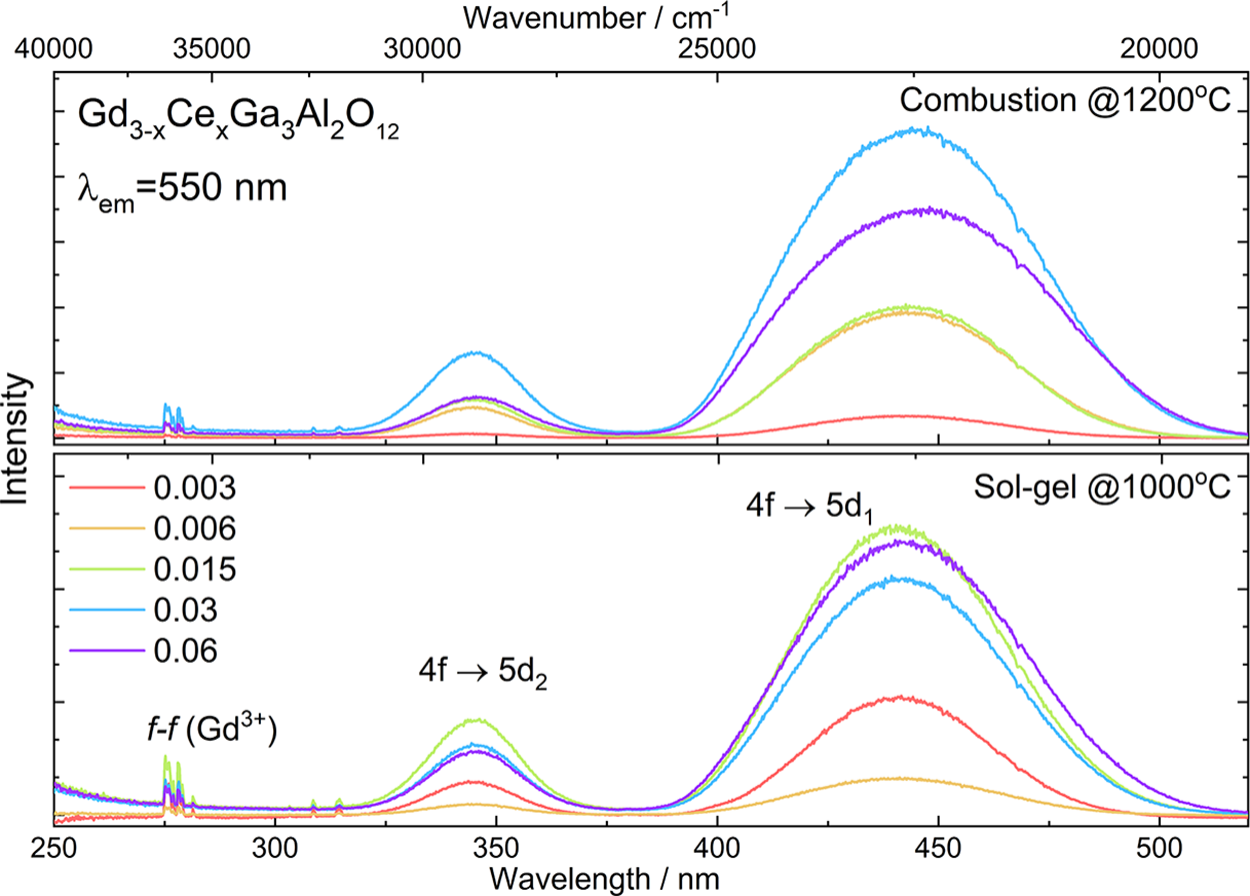
Excitation spectra of Gd_3–*x*_Ce_*x*_Ga_3_Al_2_O_12_ obtained by combustion with additional calcination (top) and Pechini sol–gel (bottom) methods.

For the powders obtained with both methods, the excitation spectra were measured at λ_em_ = 550 nm (Figure 4). Two broad bands observed in the spectra of all samples at 340 and 440 nm can be attributed to transitions from the 4f ground level of Ce^3+^ to the lowest 5d_2_ and 5d_1_ states,^47^ respectively. Sharp peaks at 275, 308, and 314 nm were attributed to the transitions from the ^8^S_7/2_ ground level to ^6^I_J_, ^6^P_3/2_, and ^6^P_7/2_ excited levels of Gd^3+^ ions, respectively.^34,48^ The presence of these peaks in the excitation spectra shows that Gd^3+^ ions absorb part of the energy in the UV range and transfer it to the excited levels of Ce^3+^ ions. It should be noted that the intensity of Gd^3+^ f–f transitions is higher for powders obtained by the sol–gel method, indicating that the smaller unit cell favors energy transfer from matrix ions to the optically active ones.

A closer look at the excitation spectra of the powders obtained by different methods also shows other interesting differences (Table 4). For both synthesis techniques, the increase of Ce^3+^ concentration leads to the red shift of 5d_1_ band, but for the combustion method, this shift is stronger and the difference between the maxima of the 5d_2_ and 5d_1_ bands (Δ_21_) is higher (for the sol–gel powder with the lowest concentration of cerium ions, due to the low signal intensity, the emission slit was doubled to separate 5d_2_ and 5d_1_ bands and be able to calculate Δ_21_). Such behavior was already observed for Ce^3+^-doped GGAG and related to the crystal field splitting and size of the crystallites.^49^ Dorenbos^50^ has shown that the red shift in the garnet family is almost independent of the centroid shift (related to the cations binding oxygen ligands) and is proportional to the crystal field splitting caused by tetragonal distortion. The higher splitting of 5d states and larger red shift of 5d bands observed for the powders obtained by the combustion method resulted from the higher disorder of the surrounding of Ce^3+^ ions displaced from the cubic polyhedron to disordered square anti-prism (dodecahedron).^47^ In the case of the sol–gel method, the unit cell (and bond length) decreases with increasing Ce^3+^ concentration, leading to lower disorder and weaker red shift. The broadening of the band with increasing Ce^3+^ concentration suggests that as the number of optically active ions in the GGAG matrix increases, they should occupy slightly different positions.^51^

**Table 4:** Positions of the 5d Levels, Full Width at Half-Maximum (FWHM), and Differences between the 5d_1_ and 5d_2_ Levels

	5d_2_		5d_1_		
*x*	position (cm^–1^)	FWHM (cm^–1^)	position (cm^–1^)	FWHM (cm^–1^)	Δ_21_ (cm^–1^)
Combustion @1200 °C					
0.003	29044	1925	22594	2597	6451
0.006	29028	1957	22573	2953	6454
0.015	29019	2007	22578	2882	6441
0.03	28,986	2044	22502	3561	6483
0.06	28927	2019	22406	4162	6520
Sol–Gel @1000 °C					
0.003	29019	1897	22655	2440	6364
0.006	29002	1911	22655	2663	6347
0.015	28986	1977	22660	2872	6325
0.03	28969	1966	22624	2945	6344
0.06	28960	1990	22563	3172	6397

**Figure 5:**
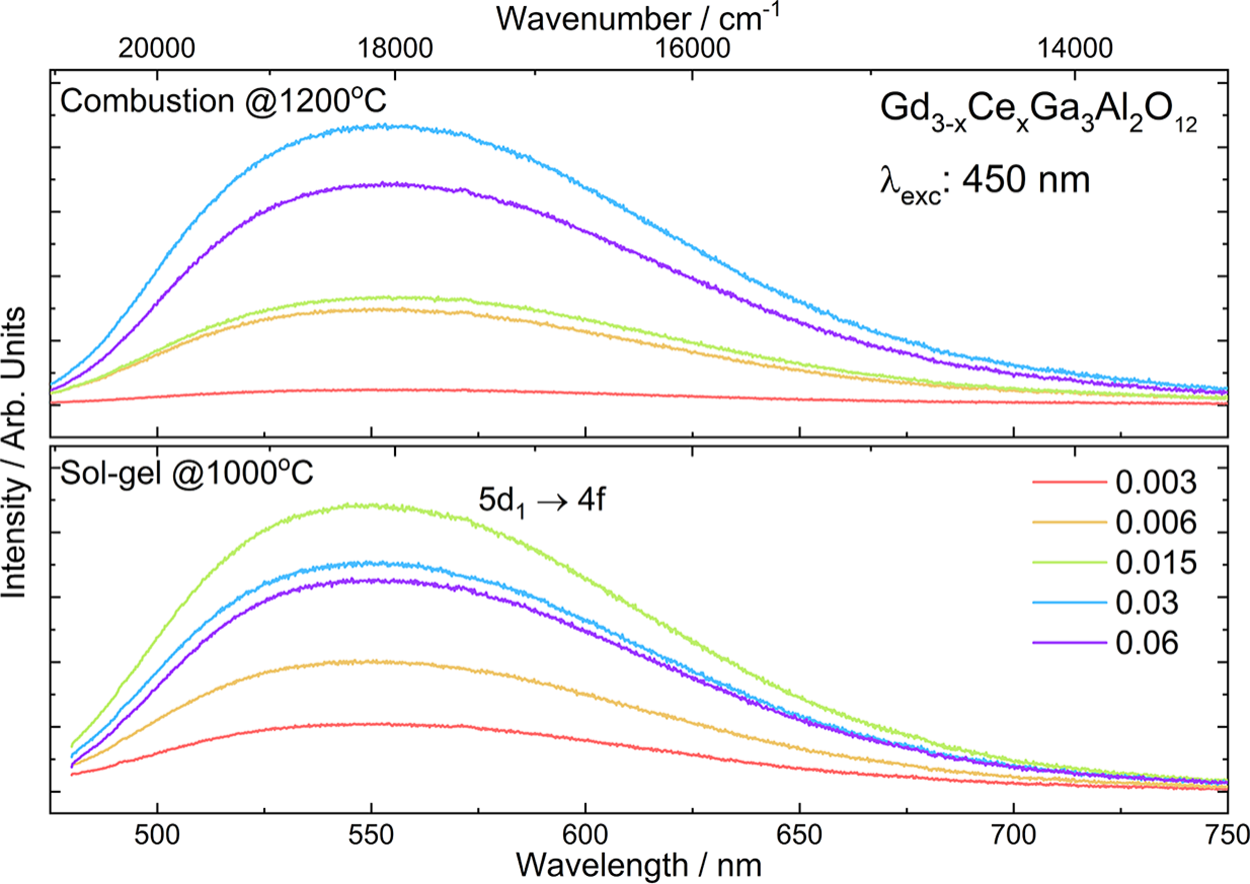
Emission spectra of Gd_3–*x*_Ce_*x*_Ga_3_Al_2_O_12_ obtained by combustion with additional calcination (top) and Pechini sol–gel (bottom) methods.

The emission spectra of the Gd_3–*x*_Ce_*x*_Ga_3_Al_2_O_12_ nanopowders were measured at room temperature using the 445 nm laser diode as an excitation source. All samples show an intense broad band centered at 550 nm corresponding to transitions from the lowest 5d_1_ level to the ^2^F_5/2_ level of Ce^3+^^52^ (Figure 5). The substitution of the Gd^3+^ by Ce^3+^ ions leads to the red shift of the luminescence band. The changes may be induced by two effects: the centroid shift (determined by the so-called nephelauxetic effect) and the crystal field splitting of the 5d orbital. The centroid shift is caused by the change of the covalency of the bond between the Ce^3+^ and the surrounding ions (in this case, oxygen anions coordinated by different cations). The second effect is the change of the Ce^3+^ crystal field splitting by interaction with the nearest neighboring ions affected by the nature of these bonds (i.e., bond length, coordination number, symmetry, etc.) leading to alteration of the spectroscopic properties of Gd_3–*x*_Ce_*x*_Ga_3_Al_2_O_12_. The crystal field splitting depends strongly on the bond lengths between luminescent ion and surrounding ligands and the type of coordination environment.^45^ As Ce^3+^ ions substituting Gd^3+^ ones in the garnet structure are located in 24(c) sites with eightfold coordination, the relation between crystal field strength and coordination environment in this case can be expressed by:
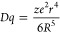
where *R* is the distance between the luminescent ion and oxygen, *z* is the charge or valence of the coordinating anions (oxygen), *e* is the charge of an electron, and *r* is the radius of the 5d wave function. From this equation, it can be seen that crystal field splitting is inversely proportional to the bond length between cerium and oxygen. In addition to the 10Dq splitting by the cubic crystal field, there is an additional splitting Δ_21_ of the higher t_2g_ state (Table 3) and the lower e_g_ state (Table 5) because of a tetragonal distortion for Ce^3+^ ions in garnets.^47^ Xia and Meijerink^45^ in their work analyzing the substitution of the cations in the garnet structures predicted that for Ce^3+^ in a larger Gd site, the increase in Ce–O distance should decrease the crystal field splitting that has been confirmed for the samples synthesized using the sol–gel method.

**Table 5:** Positions of the Emission Bands and the Differences between Them for Powders Prepared by the Combustion and Sol–Gel Methods

*x*	ν_em1_ (cm^–1^)	ν_em2_ (cm^–1^)	Δ_21_ (cm^–1^)
Combustion @1200 °C			
0.003	17603	19293	1690
0.006	17579	19252	1673
0.015	17551	19233	1682
0.03	17506	19167	1661
0.06	17461	19124	1663
Sol–Gel @1000 °C			
0.003	17854	19367	1513
0.006	17742	19332	1590
0.015	17698	19301	1603
0.03	17670	19280	1610
0.06	17612	19214	1602

For different concentrations of Ce^3+^ ions, the position of the emission band maximum changes slightly and depends on the synthesis method. It is well known that 5d → 4f Ce^3+^ transition is strongly dependent on the crystal field and emission wavelength is very sensitive to the crystallographic environment of Ce^3+^ ion. As Dorenbos^50^ has shown, the red shift observed in the emission spectra is an effect of Δ_21_ splitting of the 5d_e_ levels caused by a tetragonal distortion for Ce^3+^ in the [A] site of the cubic garnet structure. It was shown in the same paper that replacing Gd by a smaller cation (Y or Lu) leads to a decrease of the red shift and splitting of 5d-doublet levels. In the case of Gd_3–*x*_Ce_*x*_Ga_3_Al_2_O_12_ obtained by the sol–gel method, Gd^3+^ ions are substituted by larger Ce^3+^ cations, and the increase of Ce^3+^ concentration leads to an increase of the red shift and Δ_21_ splitting. It should be noted that for the samples obtained by the combustion method, the increase of Ce^3+^ concentration leads to a stronger red shift of the emission bands; however, crystal field splitting decreases. This effect is related to the phenomenon described by Ueda and Tanabe;^47^ namely, Δ_21_ can be a linear function of the lattice constant that is affected by the crystallite size and unit cell volume.^53^ Another explanation of the emission red shift with increasing Ce^3+^ concentration has a spectroscopic origin. Two effects contribute to the spectral shift: reabsorption of high energy emission of Ce^3+^ and energy transfer to distorted Ce^3+^ ions. As the absorption and emission bands overlap strongly for high Ce^3+^ concentrations, the probability for absorption of the high energy emission increases. The reabsorption leads to a decrease of the short wavelength emission intensity and red shift of the emission. The higher the number of reabsorption centers is, the larger is the red shift.^45^ At a high concentration of Ce^3+^ ions, energy transfer to neighboring distorted Ce^3+^ ions can be also observed. Excitation energy is trapped at these distorted sites, leading to emission red shift.

**Figure 6:**
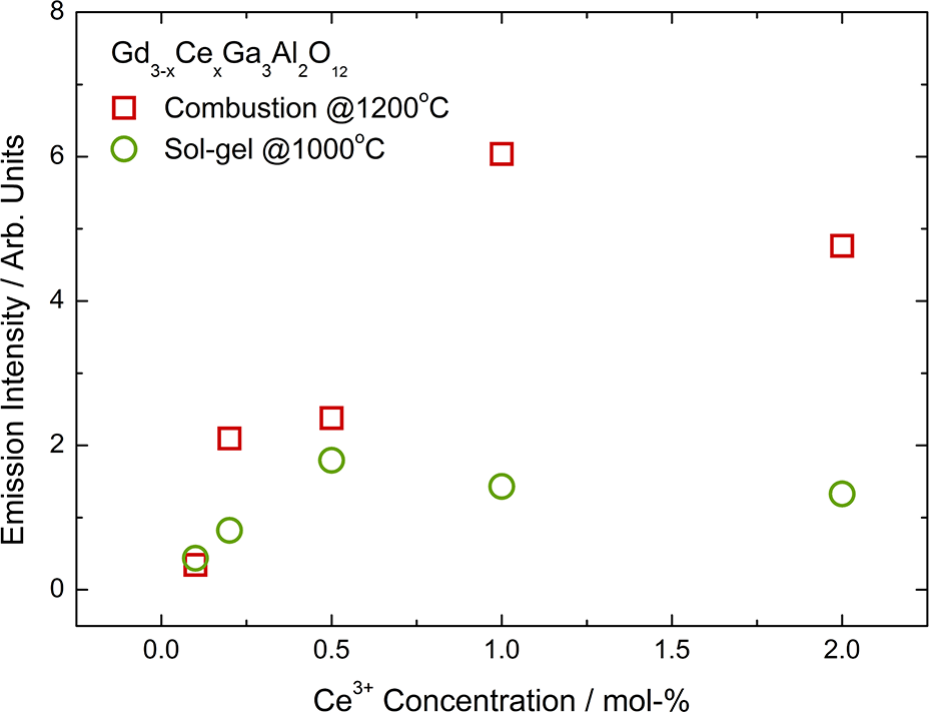
Emission intensity as a function of Ce^3+^ concentration in Gd_3–*x*_Ce_*x*_Ga_3_Al_2_O_12_ obtained by two different methods.

Emission intensity as a function of the Ce^3+^ concentration for the powders obtained by combustion and sol–gel methods is shown in Figure 6. The most intense emission for the powders obtained by the combustion method was registered for Gd_2.97_Ce_0.03_Ga_3_Al_2_O_12_ (1 mol %), and that for sol–gel samples was registered for Gd_2.985_Ce_0.015_Ga_3_Al_2_O_12_ (0.5 mol %). The values of optimal Ce^3+^ concentrations agree well with the data obtained for other Ce^3+^-doped garnets, for which the highest emission intensity was observed for the samples with 0.5–1 mol % of Ce^3+^ ions. Above this concentration, the concentration quenching is observed that can be induced by radiation reabsorption, or nonradiative de-excitation of the 5d level and recombination via the conduction band (CB) of the matrix. As the excitation band (5d_1_) partly overlaps the emission band, it is possible that part of the emission energy is reabsorbed and therefore emission is quenched. Another reason was proposed by Lesniewski et al.,^54^ who have shown using photocurrent measurements that as 5d_1_ and 5d_2_ states in GGAG overlap with CB, the electrons from excited states, regardless of temperature, can be transferred to the CB by the autoionization of Ce^3+^ leading to the quenching of Ce^3+^ emission. The powders obtained by the combustion method show higher emission intensity as they have larger grains and higher degree of crystallization (Figure 6).^55^

### Persistent Luminescence Spectra of Gd_3–*x*_Ce_*x*_Ga_3_Al_2_O_12_

**Figure 7:**
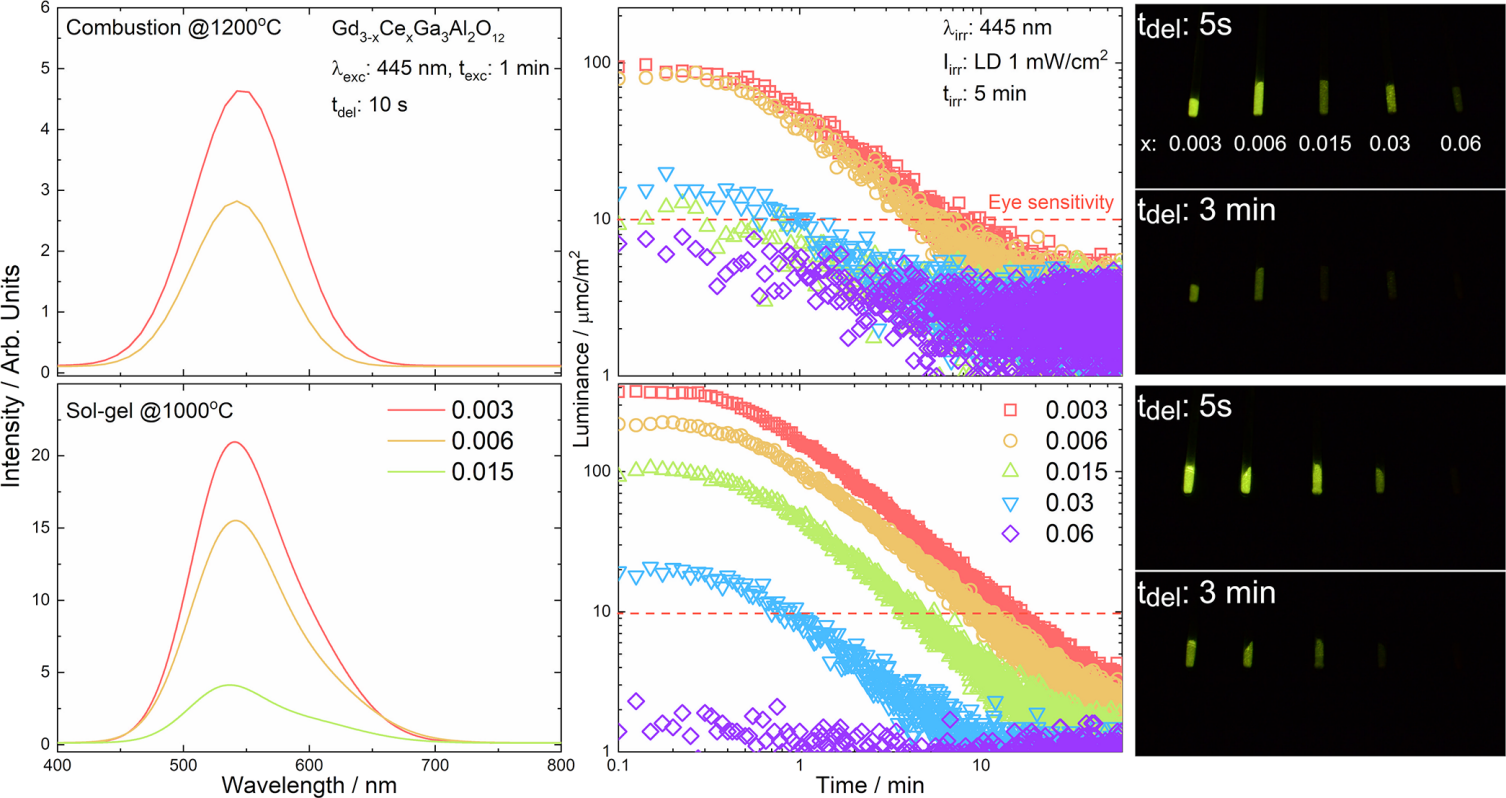
Persistent luminescence spectra (left), fading time of the emission (middle), and photo of the luminescence as a function of time after irradiation (right) for Gd_3–*x*_Ce_*x*_Ga_3_Al_2_O_12_ obtained by combustion with additional calcination (top) and Pechini sol–gel (bottom) methods.

Persistent luminescence spectra of Gd_3–*x*_Ce_*x*_Ga_3_Al_2_O_12_ powders obtained by two methods were registered after ceasing 445 nm laser diode irradiation (irradiation time was 1 min for all samples) (Figure 7). Persistent luminescence spectra show 5d → 4f Ce^3+^ transitions with the maxima corresponding to the maxima observed in conventional luminescence spectra. Similar to the conventional luminescence, spectra of the powders obtained by combustion method are red shifted. The photo and the spectra of persistent luminescence show that the most intense emission is observed for the samples doped with the lowest Ce^3+^ concentration. This behavior is observed for powders obtained by both methods. The most intense emission observed for the lowest dopant concentration is related to the low temperature needed for Ce^3+^ autoionization in case of GGAG and fast recombination of the electrons from optical centers with CB. For the powders obtained using combustion method, it was possible to register the spectra only for the two lowest Ce^3+^ concentrations, and for sol–gel samples, the spectra for the three lowest Ce^3+^ concentrations were registered. At the same time, for the higher Ce^3+^ concentration, it was not possible to register persistent luminescence spectra.

Decay times of persistent luminescence show that the most intense and longest persistent luminescence is observed for the sample with the lowest dopant concentration (Figure 6, middle). Decay is non-exponential, so the curves were fitted using a bi-exponential formula. Accordingly, at least two types of the traps are present in the Gd_3–*x*_Ce_*x*_Ga_3_Al_2_O_12_. The shallow traps release electrons faster (high brightness of persistent luminescence on the beginning of the process), while deeper traps need more energy for releasing the electrons, so these carriers are released more slowly (lower brightness, longer fading time). The fading times calculated from emission decay curves are presented in Table 6. It can be observed that for both methods, the duration of the persistent luminescence decreases with the increase of Ce^3+^ concentration. For the lowest dopant concentration, it is possible to observe persistent luminescence about 5 min after ceasing irradiation.

**Table 6:** Persistent Luminescence Decay Times Calculated for Gd_3–*x*_Ce_*x*_Ga_3_Al_2_O_12_ Powders Obtained via Different Synthesis Methods

*x*	τ_1_ (s)	τ_2_ (s)
Combustion @1200 °C		
0.003	50	331
0.006	46	301
0.015	40	194
0.03	37	203
0.06	23	130
Sol–Gel @1000 °C		
0.003	61	395
0.006	50	325
0.015	44	224
0.03	47	226
0.06	30	165

### Thermoluminescence (TL) of Gd_3–*x*_Ce_*x*_Ga_3_Al_2_O_12_

**Figure 8:**
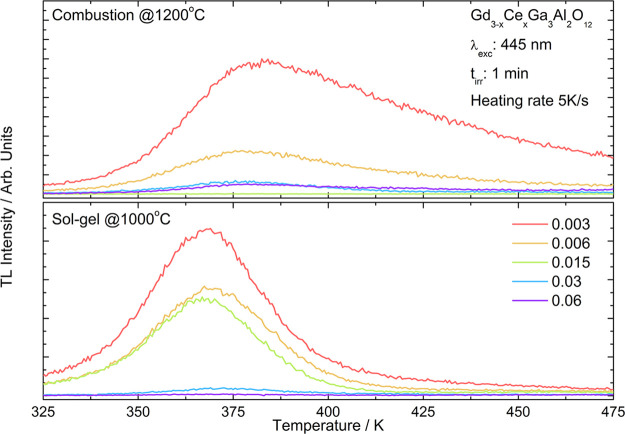
Thermoluminescence glow curves registered for Gd_3–*x*_Ce_*x*_Ga_3_Al_2_O_12_ obtained by combustion with additional calcination (top) and Pechini sol–gel (bottom) methods.

The thermoluminescence was measured for powders obtained by the two methods after irradiation by the 445 nm laser diode for 1 min. Then the samples were transferred to the measurement chamber, where TL glow curves were registered. The TL glow curves consist of a non-uniformly widened band that can be fitted using three peaks in case of powders obtained by the combustion method and two peaks in case of samples prepared by the sol–gel method (Figure 8).

The analysis of measured TL glow curves and estimation of the trap depths were performed using the GlowFit software.^56^ Because the TL peaks are first kinetic order and are not solvable analytically, the GlowFit software uses several different approximations and functions to describe them. The following expression^57^ was used to describe a single glow peak:
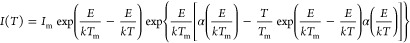
where *I* is the glow peak intensity; *k* is the Boltzmann constant; *I*_m_ and *T*_m_ are the intensity and temperature of the maximum, respectively; α is a quotient of fourth-order polynomial; and *E* is an activation energy. The positions of the maxima of the glow curves and activation energies calculated for Gd_3–*x*_Ce_*x*_Ga_3_Al_2_O_12_ samples are presented in Table 7. For the samples Gd_2.985_Ce_0.015_Ga_3_Al_2_O_12_ (combustion @1200 °C), the TL signal was too weak to calculate the activation energy.

**Table 7:** TL Glow-Curve Parameters Calculated for Gd_3–*x*_Ce_*x*_Ga_3_Al_2_O_12_ Powders Obtained via Different Synthesis Methods

*x*	*T*_m_ (K)	*E* (eV)
Combustion @1200 °C		
0.003	355.2	0.50
	382.6	0.50
	441.7	0.67
0.006	354.9	0.50
	388.8	0.61
	422.8	0.57
0.015	–	–
0.03	351.9	0.50
	363.5	0.51
	420.8	0.74
0.06	353.7	0.50
	375.1	0.53
	415.3	0.77
Sol–Gel @1000 °C		
0.003	342.7	0.51
	357.5	0.79
0.006	342.9	0.60
	355.4	0.80
0.015	341.3	0.63
	352.2	0.82
0.03	346.3	0.50
	363.6	0.86
0.06	341.5	0.63
	369.4	0.81

Analyzing this table and Figure 8, it can be seen that in the case of powders obtained by the sol–gel method, the thermoluminescence curves can be fitted with a smaller number of peaks (which means lower number of traps) and thermoluminescence is observed at lower temperatures (shallow traps). So, the traps are closer to the conduction band and less energy is needed to release the electrons from the trap and observe the persistent luminescence.

### The X-ray Photoelectron Spectroscopy (XPS) Analysis of Gd_3–*x*_Ce_*x*_Ga_3_Al_2_O_12_

**Figure 9:**
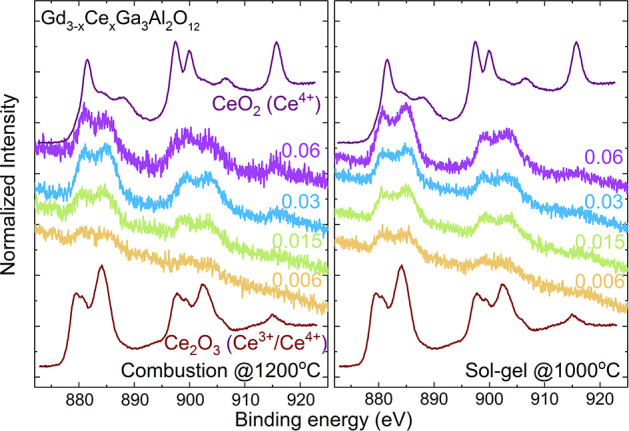
High-resolution XPS spectra of the Ce3d region recorded for Gd_3–*x*_Ce_*x*_Ga_3_Al_2_O_12_ obtained by two different synthesis methods.

Wang et al. show that the Ce^3+^/Ce^4+^ ratio in the garnet has a great impact on the luminescence efficiency.^58^ To check the valence state of the cerium ions in Gd_3–*x*_Ce_*x*_Ga_3_Al_2_O_12_, the XPS spectrum was measured and analyzed. The designation of the Ce chemical state in garnets is a complicated question because of the hybridization between Ce4f and O2p states. For the XPS spectra of Gd_3–*x*_Ce_*x*_Ga_3_Al_2_O_12_, it was assumed that the peak at ∼915 eV is assigned to the presence of the Ce^4+^ in the compound.^59^ Since the ratio of the area of high energy peak (∼915 eV) to the area of the rest of the peaks is 14:86,^60^ it is possible to roughly estimate (with an error of about 15%) the amount of Ce^4+^ in the compounds. The results were also compared to the XPS spectrum of Ce_2_O_3_ where Ce^4+^ is estimated for ∼20–30%. As the concentration of the cerium in the garnet is very low and the spectra are noisy, it is quite difficult to extract this high energy peak, but looking at the peak intensity at 915 eV and the peak intensity ratios ∼881(4+) to ∼884(3+) eV and ∼897(4+, 3+) to ∼902(3+) eV, it can be seen that the amount of Ce^4+^ is at very low level (Figure 9). It is also worth to notice that the sol–gel method promotes the reduction of the cerium ions (the ∼915 peak is less pronounced, and the peaks at ∼884 and ∼902 eV are more intense) and their incorporation into the lattice. The results of the rough calculation of the Ce^3+^ to Ce^4+^ ratio are shown in Table 8.

**Table 8:** Relative Proportions of Ce^3+^ and Ce^4+^ as Functions of Ce Concentration Calculated from XPS Spectra of Gd_3–*x*_Ce_*x*_Ga_3_Al_2_O_12_

*x*	Ce^3+^ (%)	Ce^4+^ (%)
Combustion @1200 °C		
0.006	96.46	3.54
0.015	95.82	4.18
0.03	96.40	3.60
0.06	96.56	3.44
Sol–Gel @1000 °C		
0.006	95.24	4.76
0.015	96.39	3.61
0.03	97.51	2.49
0.06	96.49	3.51

### Mechanism of Persistent Luminescence

The Ce^3+^ dopant has the same valence as the regular Gd^3+^ ion, so no charge compensation is required and energy traps should be related to other intrinsic defects. As the powders were calcinated at high temperature in air atmosphere, the presence of oxygen vacancies (V_O_^••^) acting as the traps for electrons can be assumed. The vacancies (V_O_^••^) with +2 effective charge can capture electrons from the CB and form localized negatively charged defects. Therefore, in this case, the oxygen vacancies are favorable defects. The activation energies calculated from TL glow curves are in the range from 0.5 to 0.86 eV (below the CB), so these defects are able to capture and release the electrons at room temperature. Thus, these defect levels are supposed to act as electron traps leading to the persistent luminescence.

**Figure 10:**
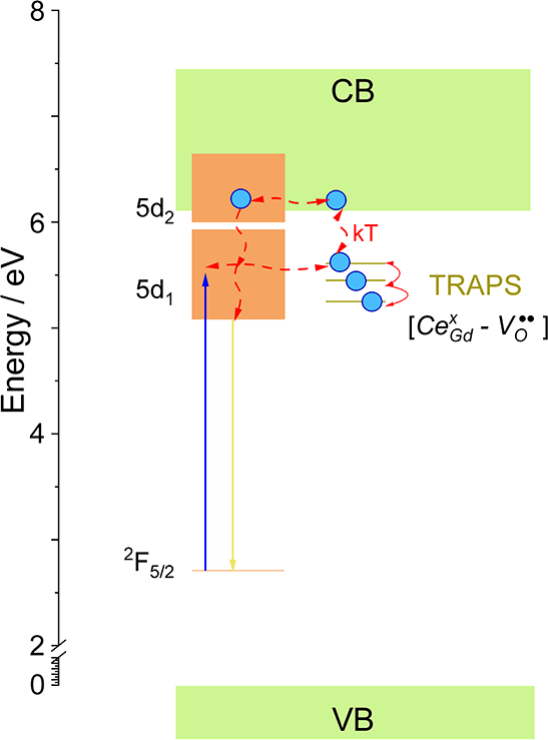
Persistent luminescence mechanism of Gd_3–*x*_Ce_*x*_Ga_3_Al_2_O_12_.

Based on the results obtained in this work, the mechanism of persistent luminescence in Gd_3–*x*_Ce_*x*_Ga_3_Al_2_O_12_ can be constructed (Figure 10). Under blue irradiation (445 nm), the electrons are excited from the ground states of Ce^3+^ ions (^2^F_5/2_) to the 5d excited levels. Part of the electrons returns to the ground state emitting yellow-green light, while another part is transferred to the V_O_^••^ where are trapped. After the cease of the excitation, the electrons captured in the shallow traps are thermally released to CB and captured by Ce^3+^ ions again. Part of the released electrons may be also transferred directly to 5d levels of Ce^3+^ ions through the tunneling processes. The released electrons captured again by Ce^3+^ ions relax to the lowest 5d_1_ level, leading to persistent luminescence. Interestingly, no persistent luminescence was observed under UV excitation, suggesting that 5d levels excited this way recombine directly with yellow-green emission and no electrons are trapped by V_O_^••^. It worth noting that the highest persistent luminescence intensity and longest fading time were observed for the powder with the lowest Ce^3+^ concentration that can be explained by two possible effects. First, when the concentration of the Ce^3+^ increases, the reabsorption process takes place and the energy that should be trapped is transferred to the another luminescent center and emitted during the conventional luminescence process. Second, for higher concentrations of Ce^3+^ ions, the probability of their presence near the traps increases, which can lead to their faster emptying, consequently reducing persistent luminescence intensity and time. In both cases, it can be assumed that as the concentration of the Ce^3+^ ions increases, the number of relaxation centers for the de-trapping process increases.

## Conclusions

The impact of different synthesis methods on the structure of Gd_3–*x*_Ce_*x*_Ga_3_Al_2_O_12_ powders was studied. The synthesis method has a big impact on the structural parameters of the crystallites (grain sizes, unit cell parameters, strain change, and bond length depend on the level of dopant). Depending on the synthesis method, the unit cell can either expand or contract with the increase of Ce^3+^ concentration, leading to the change of the distances between Ce^3+^ ions and oxygen ligands changing the spectroscopic properties of the powders. The red shift of the 5d_1_ band as well as the splitting of 5d levels with increasing Ce^3+^ concentration in the excitation spectra is determined by the change of the crystal field splitting caused by a tetragonal distortion for Ce^3+^ ions in garnets. For both methods, a broad band was observed in the emission spectra with the maximum at 550 nm originated from the transition from the lowest 5d_1_ state to the ^2^F_5/2_ ground level. It is also worth to notice that the synthesis method changed the position of the emission band maximum from 552.6 nm in the case of sol–gel synthesis to 553.2 nm in powders obtained with the combustion method due to the change of crystallographic environment and crystal field strength. The conventional emission was most intense for the samples with 1 and 0.5 mol % of Ce^3+^ ions obtained by the combustion and sol–gel method, respectively. The persistent luminescence spectra show the same emission band as conventional ones, but in this case, the longest and most intense emission was observed for the lowest Ce^3+^ concentration. This effect is observed due to the increase of the number of relaxation centers near electron traps. Because of that, it was not possible to register persistent luminescence spectra for highly doped samples. The glow curves show that at least two types of traps are present in the powder. It was also shown that the number and location of the traps are strongly affected by the synthesis method.
